# Risk of hypothyroidism in meat-eaters, fish-eaters, and vegetarians: a population-based prospective study

**DOI:** 10.1186/s12916-025-04045-7

**Published:** 2025-05-07

**Authors:** Catharina J. Candussi, William Bell, Alysha S. Thompson, Sven Knüppel, Martina Gaggl, Martin Světnička, Jan Gojda, Aedín Cassidy, Cornelia Weikert, Reynalda Córdova, Tilman Kühn

**Affiliations:** 1 Department of Nutritional Sciences, University of Vienna, Vienna, Austria; 2 Center for Public Health, Medical University of Vienna, Vienna, Austria; 3 Vienna Doctoral School of Pharmaceutical, Nutritional and Sport Sciences, University of Vienna, Josef-Holaubek-Platz 2, 1090 Vienna, Austria; 4 Co-Centre for Sustainable Food Systems & The Institute for Global Food Security, Queen’s University Belfast, Northern Ireland, UK; 5 Department Food Safety, Unit “Human Study Centre Consumer Health Protection”, German Federal Institute for Risk Assessment, Berlin, Germany; 6 Centre for Research on Diabetes Metabolism, and Nutrition of Third Faculty of Medicine, Charles University, Prague, Czech Republic; 7 Department of Internal Medicine, University Hospital Královské Vinohrady and Third Faculty of Medicine, Charles University, Prague, Czech Republic; 8 Department of Paediatrics, University Hospital Královské Vinohrady and Third Faculty of Medicine, Charles University, Prague, Czech Republic

**Keywords:** Plant-based diets, Hypothyroidism, Vegan, Vegetarian, Thyroid, Iodine

## Abstract

**Background:**

Plant-based diets are gaining popularity due to their well-documented cardiometabolic benefits and environmental sustainability. However, these diets are often lower in specific micronutrients such as iodine, raising concerns about their potential impact on thyroid health. Therefore, we examined the associations between plant-based diets and the risk of hypothyroidism.

**Methods:**

We analysed data from the UK (United Kingdom) Biobank cohort. Multivariable Cox proportional hazards regression models were used to estimate hazard ratios (HRs) and 95% confidence intervals (95% CIs) for incident hypothyroidism across vegans, vegetarians, pescatarians, poultry-eaters, low meat-eaters, and high meat-eaters aged 40–69 years. Ancillary to this, we carried out logistic regression analyses to evaluate associations between the diet groups and prevalent hypothyroidism according to International Classification of Diseases (ICD) codes at baseline.

**Results:**

We included 466,362 individuals from the UK Biobank, of which 220,514 followed a high meat, 221,554 a low meat, 5242 a poultry-based, 10,598 a pescatarian, 8057 a vegetarian, and 397 a vegan diet. During a median SD (Standard Deviation) follow-up of 12.7 (± 3.2) years, 10,831 participants developed hypothyroidism. In multivariable Cox regression models without adjustment for body mass index (BMI), none of the diets were significantly associated with the risk of hypothyroidism. However, there was a tendency for a higher risk of hypothyroidism among vegetarians compared to people following a high meat diet (HR = 1.13, 95% CI 0.98–1.30). After controlling for BMI, a potential collider, the association for vegetarians (HR = 1.23, 95% CI 1.07–1.42) became stronger and statistically significant. Furthermore, we observed a positive association between low meat-eaters (OR = 1.05, 95% CI 1.03–1.08), poultry-eaters (OR = 1.15, 95% CI 1.04–1.28), pescatarians (OR = 1.10, 95% CI 1.01–1.19) and vegetarian (OR = 1.26, 95% CI 1.15–1.38) with hypothyroidism prevalence.

**Conclusions:**

In the present study, we found a moderately higher risk of hypothyroidism among vegetarians, after controlling for BMI, a potential collider. This slightly higher risk of hypothyroidism among vegetarians requires further investigation, taking iodine status and thyroid hormone levels into account.

**Supplementary Information:**

The online version contains supplementary material available at 10.1186/s12916-025-04045-7.

## Background

According to market research, plant-based diets are gaining in popularity, with 4% of Europeans identifying as pescatarian (who eat fish or shellfish but not any other kind of meat), and 5% as vegetarians (who do not eat meat or fish), while 3% stated that they followed a vegan (who do not eat any kind of animal products) diet [1]. Reasons for choosing a more plant-based type of diet include ethical considerations such as animal rights and the environmental impact, as well as their potential health benefits [2]. Previous research has shown that plant-based diets are associated with lower risks of chronic diseases such as type 2 diabetes [3], total cancer [4], cardiovascular disease [5] as well as all-cause mortality [6, 7]. Potential health benefits from plant-based diets are particularly noticeable when these are composed of high-quality foods and low in snacks, sugary drinks and ultra-processed foods [6, 8].

Despite potential health benefits, individuals who consume strict plant-based diets may also be at risk of critically low intakes of essential nutrients such as zinc, iron, selenium, vitamin B12, or iodine [9–11]. These micronutrients are implicated in thyroid hormone regulation and production and may affect thyroid health [12, 13]. Iodine is essential for thyroid function, playing a crucial role in thyroid hormone biosynthesis [14]. According to the WHO (World Health Organization), a sufficient iodine status should be achieved with a daily intake of 150 µg, although a higher intake of 200 µg/day is recommended for pregnant and lactating women [15]. An insufficient iodine intake has detrimental effects on thyroid function, which can lead to goitre, thyroid nodules, and hypothyroidism [16]. In addition to lower intakes, the consumption of certain cruciferous vegetables (e.g. cauliflower or kale) as well as soy products as part of plant-based diets may reduce iodine bioavailability due to goitrogenic compounds in these foods [17, 18].

Iodine deficiency is one of the main causes of thyroid disease, including hypothyroidism, in regions with insufficient iodine intake [19]. In Europe, hypothyroidism affects approximately 3% of the population, with an estimated 5% of cases remaining undiagnosed [20]. Universal salt iodization programs have been successful in improving iodine intake in many countries. In regions such as the United Kingdom (UK), where salt is not routinely iodised, dietary iodine primarily comes from animal products, particularly milk and dairy, as well as seafood, fish and seaweed [21–23]. However, iodine levels in milk can vary significantly due to factors such as season, soil composition, farming practices and processing methods [24]. As the global trend shifts towards more plant-based diets, the risk of inadequate iodine intake may increase [25].

Given the limited research on plant-based diets and hypothyroidism, along with the higher risk of iodine insufficiency and increased consumption of potentially goitrogenic foods among individuals following these diets, this study aimed to assess the risk of hypothyroidism across different dietary groups (high meat-eaters, low meat-eaters, poultry-eaters, fish-eaters, vegetarians and vegans) using data from the population-based UK Biobank. We hypothesised that more plant-based diets would be associated with a higher risk of hypothyroidism due to lower iodine intake, particularly given in the UK iodine fortification is not mandatory.

## Methods

### Study population

The present analyses are based on data from the UK Biobank, a prospective cohort study comprised of over 500,000 participants aged between 40 and 69 years in the UK. The cohort was conducted between 2006 and 2010 in 22 different assessment centres across England, Scotland and Wales. During the baseline assessment, participants completed a comprehensive touchscreen questionnaire covering details on lifestyle, socioeconomic status and health status as well as 29 items on the frequency of the consumption of certain foods. Additionally, trained professionals conducted physical measurements, and participants provided biological samples. A detailed description of the UK Biobank, including details of the baseline assessment can be found elsewhere [26, 27]. Ethical approval for the UK Biobank cohort was obtained from the National Health Service North West Multi-Centre Research Ethics Committee. Written informed consent was received from all participants.

Participants with insufficient data on food frequencies at baseline (*n* = 7748) as well as participants with hypothyroidism not related to low iodine intake (*n* = 293) were excluded from the present analyses, resulting in a total of 494,144 participants eligible for inclusion in this study. For Cox models, all prevalent hypothyroidism cases (*n* = 27,782) at baseline were excluded. Furthermore, individuals with incomplete dietary data were excluded, resulting in 207,011 participants eligible for micronutrient intake analysis (e.g. iodine) (see Additional file 1: Fig. 1).

**Fig. 1:**
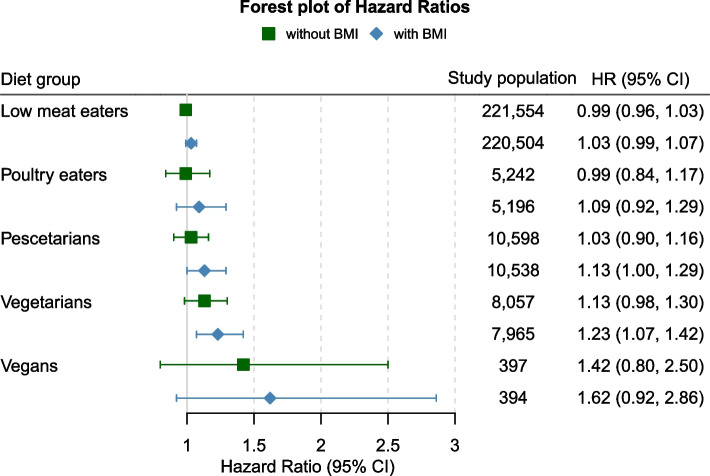
Hazard ratios (HR) and 95% Confidence intervals (95% CI) between diet groups and the risk of hypothyroidism with and without adjustment for body mass index (BMI). All models adjusted for sex, age, income, education, ethnicity, thyroid impairing medication and smoking status

### Dietary assessment and categorisation

Study participants were categorised into six different diet groups (high meat-eater, low meat-eater, poultry-eater, pescatarian, vegetarian and vegan) based on self-reported food intake from a touchscreen food frequency questionnaire completed at recruitment, as described by Bradbury et al. and Parra-Soto et al. [28, 29]. The questionnaire assessed the consumption of 29 food items, including processed meat, lamb, pork, poultry, chicken, beef, turkey, oily and non-oily fish, as well as cheese and eggs, with intake frequencies ranging from 0 (never) to 5 (once or more daily). High meat-eaters were defined as those consuming processed or unprocessed meat (poultry, red meat) more than 5–6 times a week. Low meat-eaters were defined as those consuming processed or unprocessed meat less than 5–6 times a week but at least once a week. Poultry-eaters did not consume red or processed meat, while pescatarians excluded red or processed meat and poultry from their diet. Vegetarians did not consume red or processed meat, poultry, or fish. Lastly, the vegan group was defined as individuals who “never” consumed any animal products (see Additional file 1: Fig. 2) [28, 29]. Detailed data on the consumption of iodine-rich foods and iodine intake was obtained through the validated online Oxford WebQ 24-h dietary questionnaire [30] for a subgroup of 207,011 study participants.

### Case ascertainment

Only potential diet- or low iodine intake related cases of hypothyroidism were used as endpoints for the present study using the UK Biobank’s hospital inpatient data linkage. These cases were identified using the International Classification of Diseases (ICD) codes: the 9 th edition (ICD- 9) code 244, representing ‘Acquired Hypothyroidism’, and the 10 th edition (ICD- 10) codes E01, E02, and E03.9, corresponding to ‘Iodine-deficiency related thyroid disorders and allied conditions’, ‘Subclinical Iodine-Deficiency Hypothyroidism’, and ‘Unspecified Hypothyroidism’, respectively. Hypothyroidism cases were defined as incident when occurring after baseline assessment and as prevalent when occurring before or at baseline assessment. Furthermore, individuals taking the following thyroid medication at baseline also classified as prevalent cases: levothyroxine sodium, liothyronine, sodium liothyronine, t3 – liothyronine, sodium thyroxine, thyroxine sodium, triiodothyronine 20 µg/ml injection, thyroxine product [31]. Hypothyroidism cases due to ICD- 9 code 240 (simple and unspecified goitere), 241 (nontoxic nodular goitere), 243 (congenital hypothyroidism) and ICD- 10 code E00 (congenital iodine-deficiency syndrome), E03.0-E03.8 (other hypothyroidism), E04 (other nontoxic goitres) and E06 (thyroiditis e.g. autoimmune thyroiditis or drug-induced thyroiditis) were not included in the main analysis. Nevertheless, a sensitivity analysis was conducted including all cases of hypothyroidism, as identified by the broader ICD- 9 codes 240–279, which encompass ‘Endocrine, nutritional, and metabolic diseases’ and ‘Immunity disorders’, as well as ICD- 10 codes E00–E07, which cover ‘Disorders of the thyroid gland’.

### Covariate assessment

Covariate information from baseline assessments was used as follows: Age (continuous: years), sex (categorical: male, female), ethnicity (categorical: Asian, Black, Mixed, White, Unknown), body mass index (continuous: kg/m^2^), average income (categorical: < £18,000, £18,000–£30,999, £31,000–£51,999, £52,000–£100,000, > £100,000, unknown), education (categorical: low, medium, high, unknown), smoking status (categorical: never, previous, current), thyroid impairing medication (categorical: no, yes; including data on the following drugs: amiodarone, carbamazepine product, diethylcarbamazine, carbamazepine, clozapine, quetiapine, lithium, interferon alfa 2a, interferon alfa 2b, interferon alfa, peginterferon alfa 2a, Peginterferon alfa 2b). A table of all covariates and their respective coding and item number from the UK Biobank’s online showcase can be found in Additional file 1: Table 1.

**Table 1:** Demographic characteristics of incident hypothyroidism cases

		**Overall (***N* = 466,362)	**Non- cases (***N* = 455,531)	**Cases (***N* = 10,831)
Diet	High meat eaters	220,514/466,362 (47.3%)	215,786/455,531 (47.4%)	4728/10,831 (43.7%)
	Low meat eaters	221,554/466,362 (47.5%)	216,073/455,531 (47.4%)	5481/10,831 (50.6%)
	Poultry eaters	5242/466,362 (1.1%)	5101/455,531 (1.1%)	141/10,831 (1.3%)
	Pescatarians	10,598/466,362 (2.3%)	10,342/455,531 (2.3%)	256/10,831 (2.4%)
	Vegetarians	8057/466,362 (1.7%)	7844/455,531 (1.7%)	213/10,831 (2.0%)
	Vegans	397/466,362 (0.1%)	385/455,531 (0.1%)	12/10,831 (0.1%)
Sex	Female	245,810/466,362 (52.7%)	238,102/455,531 (52.3%)	7708/10,831 (71.2%)
	Male	220,552/466,362 (47.3%)	217,429/455,531 (47.7%)	3123/10,831 (28.8%)
Age		56.41 (8.11)	56.36 (8.12)	58.33 (7.68)
Ethnicity	White	440,650/466,361(94.5%)	430,399/455,530 (94.5%)	10,251/10,831 (94.6%)
	Mixed	2761/466,361 (0.6%)	2706/455,530 (0.6%)	55/10,831 (0.5%)
	Asian	10,054/466,361 (2.2%)	9771/455,530 (2.1%)	283/10,831 (2.6%)
	Black	7268/466,361 (1.6%)	7145/455,530 (1.6%)	123/10,831 (1.1%)
	Unknown	5629/466,361 (1.2%)	5510/455,530 (1.2%)	119/10,831 (1.1%)
Average Income	Greater than 100,000	22,085/466,362 (4.7%)	21,822/455,531 (4.8%)	263/10,831 (2.4%)
	52,000 to 100,000	82,604/466,362 (17.7%)	81,330/455,531 (17.9%)	1274/10,831 (11.8%)
	31,000 to 51,999	104,910/466,362 (22.5%)	102,863/455,531 (22.6%)	2047/10,831 (18.9%)
	18,000 to 30,999	100,651/466,362 (21.6%)	98,174/455,531 (21.6%)	2477/10,831 (22.9%)
	Less than 18,000	88,193/466,362 (18.9%)	85,439/455,531 (18.8%)	2754/10,831 (25.4%)
	Unknown	67,919/466,362 (14.6%)	65,903/455,531 (14.5%)	2016/10,831 (18.6%)
Education	High	220,046/466,362 (47.2%)	215,664/455,531 (47.3%)	4382/10,831 (40.5%)
	Medium	84,778/466,362 (18.2%)	82,913/455,531 (18.2%)	1865/10,831 (17.2%)
	Low	153,454/466,362 (32.9%)	149,144/455,531 (32.7%)	4310/10,831 (39.8%)
	Unknown	8084/466,362 (1.7%)	7810/455,531 (1.7%)	274/10,831 (2.5%)
Smoking status	Current	51,258/487,524 (10.5%)	48,841/463,391 (10.5%)	2417/24,133 (10.0%)
	Previous	168,382/487,524 (34.5%)	159,317/463,391 (34.4%)	9065/24,133 (37.6%)
	Never	266,211/487,524 (54.6%)	253,658/463,391 (54.7%)	12,553/24,133 (52.0%)
	Unknown	1673/487,524 (0.3%)	1575/463,391 (0.3%)	98/24,133 (0.4%)
BMI (kg/m^2^)		27.35 (4.74)	27.33 (4.72)	28.28 (5.33)
	Unknown	2,440	2,351	89
Physical activity	High	112,200/466,362 (24.1%)	109,792/455,531 (24.1%)	2408/10,831 (22.2%)
	Moderate	183,815/466,362 (39.4%)	179,983/455,531 (39.5%)	3832/10,831 (35.4%)
	Low	66,297/466,362 (14.2%)	64,668/455,531 (14.2%)	1629/10,831 (15.0%)
	Unknown	104,050/466,362 (22.3%)	101,088/455,531 (22.2%)	2962/10,831 (27.3%)
Thyroid impairing drugs		1668/466,362 (0.4%)	1544/455,531 (0.3%)	124/10,831 (1.1%)

Categorical variables are expressed as the number of cases divided by the total number of observations (*n*/*N*), followed by the percentage in parentheses: *n*/*N* (%). Continuous variables are expressed as the arithmetic mean and standard deviation (mean ± (SD))

Abbreviation: *BMI*, body mass index (calculated as weight in kilogrammes divided by height in metres squared)

### Statistical analysis

Descriptive characteristics were assessed across six different diet groups (high meat-eaters, low meat-eaters, poultry-eaters, pescatarians, vegetarians and vegans) and shown as percentages for categorical variables and means ± standard deviations (SD) for continuous variables. To analyse the risk of hypothyroidism across the different diet groups, Cox proportional hazards regression models were used with age as the underlying time variable. The R packages ‘survival’ and ‘survminer’ were used to perform these analyses [32, 33].

Covariates for statistical adjustment were selected following a comprehensive literature search on potential risk factors for hypothyroidism. Based on literature by Chaker et al. [16], Lawton et al. [34], Tonstad et al. [35] and Rizzo et al. [36], our Cox models were adjusted for the following variables: age, sex, ethnicity, average income, educational level, thyroid impairing medication and smoking status. Initially, alcohol intake and physical activity were also included but as they did not significantly improve model fit and only marginally affected the main associations, we excluded them from the final models. According to the literature, BMI was considered a potential confounder [37, 38]. However, since hypothyroidism (possibly also before diagnosis) affects BMI through the physiological influence of thyroid hormones [39, 40], it is important to consider that BMI could be a collider, for which no adjustment should be carried out (see Additional file 1: Fig. 3). Therefore, our analyses are presented both with and without adjustment for BMI, the latter to account for potential collider bias. Furthermore, additional sensitivity analyses were conducted accounting for hypothyroidism subtypes restricting models to iodine-related hypothyroidism vs. models based on all cases of hypothyroidism (see the ‘Case ascertainment’ section).

In addition to our main prospective analyses, we conducted logistic regression analyses to investigate whether diet groups were associated with prevalent hypothyroidism, adjusting for the same set of confounders. Results were presented as hazard ratios (HRs) and 95% confidence intervals (95% CIs) for Cox models, and odds ratios (ORs) and 95% CIs for logistic regression analyses. The proportional hazards assumption was tested via the Schoenfeld residual test. No violations of proportionality were observed. Results were considered as statistically significant at a two-sided *p*-values of < 0.05. All statistical analyses were carried out using R (R version 4.3.1) [41].

## Results

### Characteristics of the study population

Baseline characteristics of the cohort are presented in Table 1. Our analysis included a total of 466,362 participants, 52.7% of whom were female, with a mean age of 56.41 ± 8.11 years. Overall, 47.3% followed a high meat diet, 47.5% a low meat diet, 1.1% a poultry diet, 2.3% a pescatarian diet, 1.7% a vegetarian diet, and 0.1% a vegan diet. During a mean follow-up period of 12.7 ± 3.2 years, 10,831 participants developed potential iodine-related hypothyroidism. The proportion of hypothyroidism cases across the diet groups was as follows: 2% among high meat-eaters, 2% among low meat-eaters, 3% among poultry-eaters, 2% among pescatarians, 3% among vegetarians, and 3% among vegans (see Additional file 1: Table 2).

**Table 2:** Odds ratios of prevalent hypothyroidism (*n* = 27,782) across diet groups in 494,144 UK-Biobank participants

	Model 1		Model 2	
Diet group	OR 95% CI	*p*-value	OR 95% CI	*p*-value
High meat eaters	Ref		Ref	
Low meat eaters	1.01 (0.98–1.04)	0.50	1.05 (1.03–1.08)	< 0.001
Poultry eaters	1.04 (0.93–1.15)	0.50	1.15 (1.04–1.28)	0.007
Pescatarians	0.98 (0.90–1.06)	0.60	1.10 (1.01–1.19)	0.022
Vegetarians	1.15 (1.05–1.26)	0.002	1.26 (1.15–1.38)	< 0.001
Vegans	0.97 (0.61–1.46)	0.9	1.15 (0.72–1.74)	0.5

Model 1 adjusted for sex, age income, education, and thyroid impairing medication. Model 2 adjusted for sex, age income, education, body mass index (BMI) and thyroid impairing medication

*OR* odds ratio, *95% CI*, confidence interval

The consumption of iodine-rich foods as well as cruciferous vegetables and soy across diet groups among the 207,011 participants with 24-h dietary data is shown in Additional file 1: Table 3. Those following a high meat, low meat, poultry, or pescatarian diet had average iodine intakes of around 200 μg/day (range: 217–204 μg/day). In contrast, those following a vegetarian or vegan diet, had mean iodine intakes of 164 (± 68) μg/day and 93 (± 45) μg/day, respectively (see Additional file 1: Table 4). As seen in Additional file 1: Table 5, about 92% of vegans, 44% of vegetarians, and 33% of poultry-eaters did not meet a sufficient daily iodine intake of 150 μg.

Overall, individuals who developed hypothyroidism were more likely to be female (71.2%), have a higher BMI 28.28 and have a lower income (see Table 1). Similar patterns were observed for prevalent hypothyroidism cases (*n* = 27,782), with the majority being females (84.0%) and from lower income groups at baseline (see Additional file 1: Table 6).

### Diets and risk of hypothyroidism

Given the potential role of BMI as a collider in model on diet a hypothyroidism (see Statistical Methods and Additional file 1: Fig. 3), multivariable associations between diet type and hypothyroidism with and without adjustment for BMI are shown in Fig. 1. None of the diet types were significantly associated with hypothyroidism risk in multivariable models without adjustment for BMI, although a marginal association was observed for vegetarians (HR = 1.13, 95% CI 0.98–1.30). After adjusting for BMI, the association for vegetarians (HR = 1.23, 95% CI 1.07–1.42) became stronger and statistically significant. In sensitivity analyses including non-iodine-related hypothyroidism cases, we observed similar results across all diet groups (see Additional file 1: Fig. 4).

Logistic regression analyses on a vegetarian diet and prevalent hypothyroidism showed a multivariable-adjusted OR (95% CI) of 1.15 (1.05–1.26) and 1.26 (1.15–1.38), respectively, after controlling for BMI (Table 2). Furthermore, greater odds for low meat-eaters (OR = 1.05, 95% CI 1.03–1.08), poultry-eaters (OR = 1.15, 95% CI 1.04–1.28) and pescatarians (OR = 1.10, 95% CI 1.01–1.19) were observed after BMI adjustment.

## Discussion

In the present study, we observed no statistically significant associations between diets lower in animal products and hypothyroidism risk in multivariable models not adjusted for BMI. After adjusting for BMI, the associations for vegetarians (HR = 1.23, 95% CI 1.07–1.42) became slightly stronger in magnitude and statistically significant. Similarly, analyses on diet types and hypothyroidism cases at recruitment (prevalent cases) showed greater odds among low meat-aters (OR = 1.05, 95% CI 1.03–1.08), poultry-eaters (OR = 1.15, 95% CI 1.04–1.28) and pescatarians (OR = 1.10, 95% CI 1.01–1.19) we compared to high meat-eaters, while no association between vegan diet and hypothyroidism were observed.

Given the influence of BMI on our results, it is crucial to consider whether BMI constitutes a collider or a confounder regarding the association between type of diet and hypothyroidism. A confounder *affects* the exposure and the outcome [42], while a collider *is influenced* by both the exposure and the outcome [43]. Statistical adjustment for confounders is necessary, whereas adjustment for colliders may bias associations. There are arguments that BMI could confound the association between diet type and hypothyroidism: A recent Mendelian randomisation analysis by Qiu et al. indicates that BMI may affect hypothyroidism, as genetically predicted BMI was associated with higher risk of hypothyroidism [44]. In turn, genetically predicted hypothyroidism was not associated with higher BMI. Considering the associations between BMI with both diet type and hypothyroidism in our study, adjustment for BMI as a confounder could be necessary. However, another recent Mendelian randomisation by Zhou et al. did not show an association between genetically predicted BMI and hypothyroidism [45]. A role of BMI as a collider seems likely, as the types of diet in our study may influence BMI rather than vice versa, given lower calorie intake among people following plant-based diets [46]. Adjustment for BMI may introduce collider bias, as it is influenced by diet, through lower caloric intake among people following a more plant-based diet, but possibly also by hypothyroidism, through the metabolic changes associated with the condition, even in its subclinical form with potential effects prior to diagnosis [47]. While collider bias seems plausible, we can only speculate that undiagnosed hypothyroidism or impaired thyroid function affected BMI in our study, as no data on thyroid health prior to BMI measurements were available. Thus, despite studies on the occurrence of unrecognised subclinical hypothyroidism among people with higher BMI, the temporality of increases in BMI and the occurrence of hypothyroidism in our study remains unclear [20, 48].

In a similar study from the United States, Tonstad et al. analysed data of 97,000 American adults enrolled in the Adventist Health Study II (AHS-II). Study participants following a vegan (OR = 0.89, 95% CI 0.78–1.01) or pescatarian (OR = 1.02, 95% CI 0.90–1.15) diet did not show a higher risk of hypothyroidism compared to omnivores whereby no data on poultry-eaters were published [8]. By contrast, a higher risk was observed for vegetarians (OR = 1.09, 95% CI 1.01–1.18) for hypothyroidism, which is in line with the present findings. Of note, the analyses of Tonstad et al. were adjusted for BMI. To our knowledge, there are no other studies on diet types in relation to hypothyroidism, although two studies, in which dietary patterns were analysed, are somewhat consistent with our findings. Alkhatib et al. observed in a cohort from three cycles of the National Health and Nutrition Examination Surveys (NHANES) that people following a ‘fat–processed grains–sugars–meats’ or an ‘oils–nuts–potatoes–low-fat meats’ dietary pattern had a lower risk of hypothyroidism compared to those with ‘fruits–whole grains–vegetables–dairy’ pattern [49]. In another cohort study from South Korea, participants were grouped by four dietary patterns (Korean balanced diet, plant-based diet (PBD), Western-style diet (WSD) and rice-based diet). The study found that individuals with a high polygenic risk and a high plant-based diet score had an increased risk of hypothyroidism [50].

Slightly higher risks of hypothyroidism among vegetarians and vegans may be explained by other mechanisms beyond suboptimal iodine intake. Vegetarians, and particularly vegans had slightly higher intakes of cruciferous vegetables compared to meat-eaters in our study. Various animal as well as human studies suggested negative effects of sulphur components of these vegetables on thyroid cell function [51–53]. However, a systematic review carried out by Galanty et al. concluded that cruciferous vegetables are generally safe for thyroid function when consumed in typical amounts, especially with adequate iodine intake, although excessive raw consumption may pose risks [54]. Compared to meat-eaters, haem iron intakes were lower among vegetarians and vegans, which may also explain slightly higher risks of hypothyroidism, given that iron deficiency can negatively affect the hypothalamic–pituitary–thyroid axis and ultimately lead to lower thyroid hormone levels [55–57]. Differences in intakes of zinc or copper may further underlie the observed associations between diet type and hypothyroidism. However, considering that higher risks of hypothyroidism among vegetarians were only observed upon statistical adjustment for BMI, collider bias remains an alternative explanation for these associations. Furthermore, the observation of greater odds of prevalent hypothyroidism among low-meaters, poultry-eaters, pescatarians and vegetarians may indicate that more health-conscious dietary choices may well be a consequence of hypothyroidism. Given that individuals with subclinical hypothyroidism often experience reduced appetite and weight gain, potentially leading them to adopt healthier dietary choices prior to diagnosis, we cannot rule out that associations between the vegetarian diet and incident hypothyroidism were in part due to reverse causation [58].

A systematic review and meta-analysis including 11 studies (*n* = 4421) showed that a vegan diet is associated with lower intakes of iodine and lower median urinary iodine concentration (12.2–44.0 µg/l) compared to omnivores [59]. According to the WHO, optimal urinary iodine levels in adults range between 100 and 200 µg/l [60]. A sufficient iodine status should be achieved with a daily intake of 150 µg, although a higher intake of 200 µg/day is recommended for pregnant and lactating women [15]. Especially in regions where universal salt iodisation programmes are absent or voluntary such as the UK, studies suggest that vegetarian and vegan diets are not appropriate to meet dietary recommendations and may increase the risk for iodine deficiency [61, 62]. This is also reflected by our results, with the majority of individuals following a regular omnivorous diet demonstrating adequate iodine intake (> 150 µg), compared to 55.6% of vegetarians and just 7.8% of vegans.

The present results on iodine intake may seem surprising, as dairy products that are consumed by vegetarians are the main source of dietary iodine in the UK [63]. However, there is wide variability in iodine levels in milk which is dependent on a range of factors including farming practices [24]. Overall, cow’s milk naturally contains a low concentration of iodine but becomes a good source of this nutrient due to typical farming practices such as using ionized disinfectant or adding iodine salts to cattle feed [64]. According to a market survey data conducted in the UK, around 20% of plant-based dairy products are fortified with iodine and iodine levels in cow milk were found to be ten times higher than those in plant-based alternatives [65]. Iodine intakes were lower among vegetarians in our study compared to meat- eaters and pescatarians, and the differences in average iodine intakes are potentially attributable to omitting fish and meat. Vegans, who had substantially lower iodine intakes compared to all other groups, did not have a higher risk of hypothyroidism, although analyses among our study were restricted to a very small number of vegans. Our finding of an only slightly greater risk of hypothyroidism among vegetarians could be partly due to the thyroid gland’s ability to adapt to mild iodine deficiency, sustaining normal thyroid hormone synthesis [66]. Henjum et al. investigated that thyroid hormone concentrations among vegans and vegetarians remain normal, despite the fact that both diets are associated with lower urinary iodine concentrations [67]. Extensive epidemiological research indicates that prolonged thyroid stimulation, resulting from this adaptive response, promotes thyroid enlargement. During this phase of follicular cell proliferation, there is an increased likelihood of mutations, which can lead to multifocal autonomous growth and subsequent thyroid dysfunction [66, 68]. Thus, further research on plant-based diets and thyroid health from population-based studies is needed to monitor potential adverse effects.

One limitation of this study is its observational design, as residual or unmeasured confounding cannot be ruled out. Despite a substantial number of hypothyroidism cases during follow-up, our analyses were not well-powered to detect moderate associations among vegans due to the relatively low number of individuals in this group. Additionally, primary care data was not available to identify hypothyroidism cases in this study. Misclassification of diet groups is also a limitation, as participants may have altered their diet during the follow-up or underreported their intake. This is reflected by the lower amounts of meat and fish consumption reported during 24-h dietary assessments by some individuals classified as vegans or vegetarians according to the baseline food frequency questionnaire. Such misclassification may have led to a potential underestimation of the risk estimates in our study. Data on iodine intake were only available for a subsample of the population and there was no data on iodine status or iodine hormone levels. No data on iodine supplementation or fortified vegan products was available leading to a potential underestimation of iodine intake, although iodine intake data is consistent with data on iodine status among people following plant-based diets from previous studies [59, 67]. Moreover, the dietary data collected from the baseline touchscreen questionnaire did not include total energy intake, so energy could not be included as a potential confounder in the multivariable models. Finally, the UK Biobank cohort represents a population with a healthier risk profile compared to the general population and includes predominantly British participants, most of whom are of white European ancestry [69, 70]. This may limit the generalizability of the findings to other populations.

## Conclusions

Overall, the findings from our study indicate that a vegetarian diet may be associated with a moderately higher risk of hypothyroidism. This finding is consistent with findings from a previous study and warrants further investigation in studies with data on iodine status and thyroid function prior to diagnosis. Given the increasing popularity of plant-based diets, it is important to consider iodine as a potential critical nutrient for vegetarians, particularly in regions without mandatory salt iodization programs. In such cases, iodine supplementation should be considered to help mitigate potential deficiencies. Additionally, more routine testing for vegetarians and vegans, with a specific focus on thyroid health, may be beneficial to identify and address potential risks at an early stage [25]. Thus, future prospective studies with more detailed data on nutrient intake and status in relation to hypothyroidism are needed.

## Supplementary Information

Supplementary Material 1: Figures S1. Flowchart. Figures S2. Dietary Categorisation. Figures S3. Illustration of collider and confounding bias. Figures S4. Hazard ratios (HR) and 95% Confidence intervals (95% CI) between diet groups and the risk of hypothyroidism with all hypothyroidism cases and cases only due to potential low iodine intakes. Table S1. List of Covariates. Table S2. Demographic characteristics of dietary groups. Table S3. Intake of different food types according to 24-h recall among diet groups within a subsample of 207,011 participants. Table S4. Average micronutrient intake across dietary groups with data from first FFQ (*n* = 207,011). Table S5. Iodine intake on whether individuals met or did not meet the recommended intake threshold of over 150 μg/day among a subsample of 207,011. Table S6. Demographic characteristics of prevalent hypothyroidism cases.

## Data Availability

The data used in this study are available from the UK Biobank (https://www.ukbiobank.ac.uk/) but are subject to restrictions. Access to these data requires a license and is not publicly available. However, the data can be obtained from the authors upon reasonable request and with permission from the UK Biobank.

## Abbreviations

AHS-II: Adventist Health Study II
BMI: Body mass index
CI: Confidence interval
HR: Hazard ratio
ICD: International Statistical Classification of Diseases and Related Health Problems
NHANES: National Health and Nutrition Examination Surveys
OR: Odds ratio
PBD: Plant-based diet
SD: Standard deviation
UK: United Kingdom
WHO: World Health Organization
WSD: Western-style diet
