# Supplementary material for: Risk of hypothyroidism in meat-eaters, fish-eaters, and vegetarians: a population-based prospective study
Source: BMC Med. 2025 May 7;23:269. doi: 10.1186/s12916-025-04045-7 (PMC12060288; doi:10.1186/s12916-025-04045-7)
Supplement: Supplementary file 1 — Supplementary Material 1: Figures S1. Flowchart. Figures S2. Dietary Categorisation. Figures S3. Illustration of collider and confounding bias. Figures S4. Hazard ratios (HR) and 95% Confidence intervals (95% CI) between diet groups and the risk of hypothyroidism with all hypothyroidism cases and cases only due to potential low iodine intakes. Table S1. List of Covariates. Table S2. Demographic characteristics of dietary groups. Table S3. Intake of different food types according to 24-h recall among diet groups within a subsample of 207,011 participants. Table S4. Average micronutrient intake across dietary groups with data from first FFQ (n = 207,011). Table S5. Iodine intake on whether individuals met or did not meet the recommended intake threshold of over 150 μg/day among a subsample of 207,011. Table S6. Demographic characteristics of prevalent hypothyroidism cases. [file 12916_2025_4045_MOESM1_ESM.docx]

**Supplementary material**

**Figure 1**: Flowchart


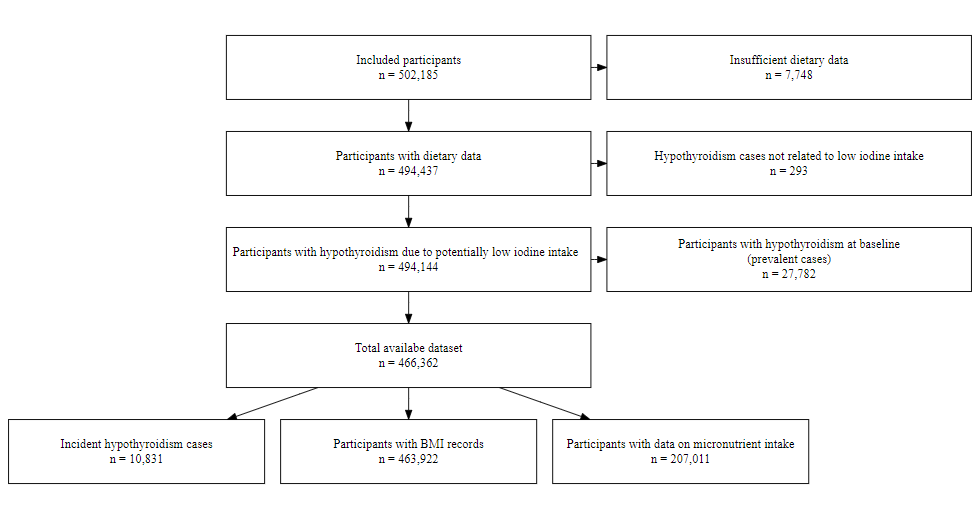


**Figure 2**: Dietary Categorisation
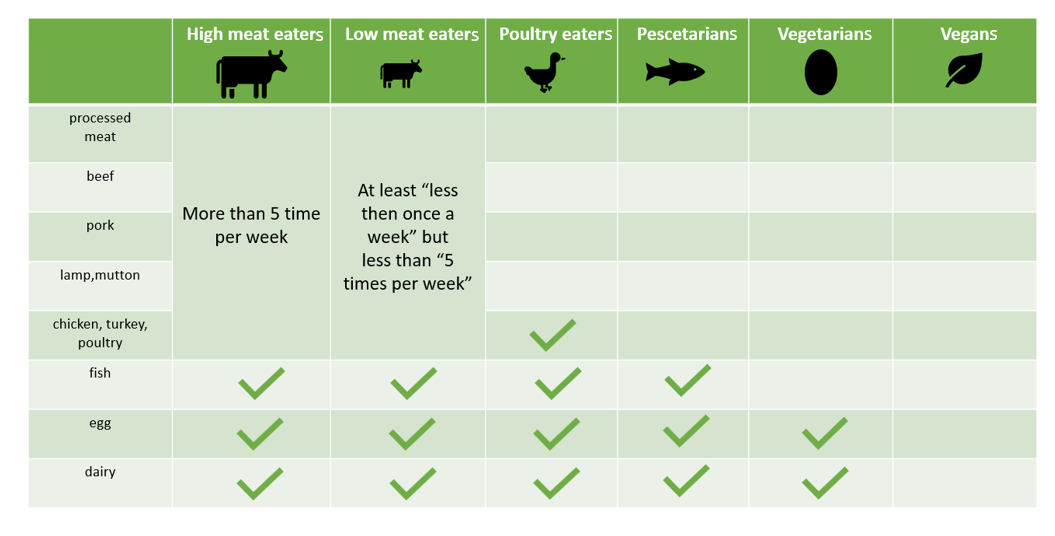


**Table 1**: List of Covariates

| **Variables** | **UK Biobank Showcase Data Field** | **Type** | **Coding** |
| --- | --- | --- | --- |
| Sex | 31 | Catagorical: male, female | NA |
| Age | 21022 | Continuous: years | NA |
| Ethnicity | 100065 | Categorical: Asian, Black, Mixed, White, Unknown | NA |
| BMI | 23104 | Continuous: kg/m^2^ | NA |
| Household Income | 738 | Catagorical: Greater than 100,000; 52,000 to 100,000; 31,000 to 51,999; 18,000 to 30,999; Less than 18,000; Unknown | NA |
| Education | 6138 | Categorical: Low, Medium, High, Unknown | Low = “None of the above”, “O-level/GCSE”, “cse or equivalent”  Medium = “A-level/AS Level”, “NVQ, HND, HNC”  High = “College or University Degree”, “Other Professional Qualification”  Unknown = „prefer not to answer“, missing, „none of the above“ |
| Smoking status | 20116 | Categorical: Never, previous, Current, Unknown | NA |
| Thyroid impairing medication | 20003 | Categorical: Yes/ no | amiodarone, carbamazepine product, diethylcarbamazine, carbamazepine, clozapine, quetiapine, lithium, interferon alfa 2a, interferon alfa 2b, interferon a  lfa, peginterferon alfa 2a, peginterferon alfa 2b |
| Thyroid medication | 20003 | Categorical: Yes/ no | levothyroxine sodium, liothyronine, sodium liothyronine, t3 – liothyronine, sodium thyroxine, thyroxine sodium, triiodothyronine 20micrograms/ml injection, thyroxine product |

**Figure 3:** Illustration of collider and confounding bias

**Table 2:** Demographic characteristics of dietary groups

|  | **High meat eaters** N = 220,514 | **Low meat eaters** N = 221,554 | **Poultry eaters** N = 5,242 | **Pescatarians** N = 10,598 | **Vegetarians** N = 8,057 | **Vegans** N = 397 |
| --- | --- | --- | --- | --- | --- | --- |
| Incident cases | 4,728 / 220,514 (2%) | 5,481 / 221,554 (2%) | 141 / 5,242 (3%) | 256 / 10,598  (2%) | 213 / 8,057  (3%) | 12 / 397  (3%) |
| Sex |  |  |  |  |  |  |
| Female | 91,548 / 220,514 (42%) | 137,327 / 221,554 (62%) | 3,923 / 5,242 (75%) | 7,523 / 10,598 (71%) | 5,261 / 8,057 (65%) | 228 / 397 (57%) |
| Male | 128,966 / 220,514 (58%) | 84,227 / 221,554 (38%) | 1,319 / 5,242 (25%) | 3,075 / 10,598 (29%) | 2,796 / 8,057 (35%) | 169 / 397 (43%) |
| Age | 56.49 (8.17) | 56.57 (8.02) | 56.32 (8.07) | 54.03 (8.05) | 53.02 (7.94) | 53.61 (7.99) |
| Ethnicity |  |  |  |  |  |  |
| White | 210,110 / 220,514 (95%) | 209,177 / 221,553 (94%) | 4,707 / 5,242 (90%) | 9,881 / 10,598 (93%) | 6,410 / 8,057 (80%) | 365 / 397 (92%) |
| Mixed | 1,233 / 220,514 (1%) | 1,328 / 221,553 (1%) | 37 / 5,242  (1%) | 95 / 10,598  (1%) | 63 / 8,057  (1%) | 5 / 397  (1%) |
| Asian | 3,436 / 220,514 (2%) | 4,648 / 221,553 (2%) | 232 / 5,242 (4%) | 315 / 10,598  (3%) | 1,419 / 8,057 (18%) | 4 / 397  (1%) |
| Black | 3,241 / 220,514 (1%) | 3,653 / 221,553 (2%) | 164 / 5,242 (3%) | 163 / 10,598  (2%) | 38 / 8,057  (0%) | 9 / 397  (2%) |
| Unknown | 2,494 / 220,514 (1%) | 2,748 / 221,553 (1%) | 102 / 5,242 (2%) | 144 / 10,598  (1%) | 127 / 8,057  (2%) | 14 / 397  (4%) |
| BMI (kg/m^2^) | 27.90 (4.80) | 27.02 (4.63) | 25.46 (4.49) | 25.20 (4.24) | 25.63 (4.57) | 24.69 (4.44) |
| Unknown | 1,189 | 1,050 | 46 | 60 | 92 | 3 |
| Average Income |  |  |  |  |  |  |
| Greater than 100,000 | 10,375 / 220,514 (5%) | 10,604 / 221,554 (5%) | 207 / 5,242 (4%) | 557 / 10,598  (5%) | 333 / 8,057  (4%) | 9 / 397  (2%) |
| 52,000 to 100,000 | 39,193 / 220,514 (18%) | 38,619 / 221,554 (17%) | 809 / 5,242 (15%) | 2,329 / 10,598 (22%) | 1,593 / 8,057 (20%) | 61 / 397 (15%) |
| 31,000 to 51,999 | 50,063 / 220,514 (23%) | 49,243 / 221,554 (22%) | 1,011 / 5,242 (19%) | 2,551 / 10,598 (24%) | 1,939 / 8,057 (24%) | 103 / 397 (26%) |
| 18,000 to 30,999 | 47,720 / 220,514 (22%) | 48,019 / 221,554 (22%) | 1,075 / 5,242 (21%) | 2,164 / 10,598 (20%) | 1,594 / 8,057 (20%) | 79 / 397 (20%) |
| Less than 18,000 | 41,624 / 220,514 (19%) | 41,960 / 221,554 (19%) | 1,279 / 5,242 (24%) | 1,781 / 10,598 (17%) | 1,437 / 8,057 (18%) | 112 / 397 (28%) |
| Unknown | 31,539 / 220,514 (14%) | 33,109 / 221,554 (15%) | 861 / 5,242 (16%) | 1,216 / 10,598 (11%) | 1,161 / 8,057 (14%) | 33 / 397  (8%) |
| Education |  |  |  |  |  |  |
| High | 99,116 / 220,514 (45%) | 105,889 / 221,554 (48%) | 2,722 / 5,242 (52%) | 7,079 / 10,598 (67%) | 4,979 / 8,057 (62%) | 261 / 397 (66%) |
| Medium | 42,490 / 220,514 (19%) | 38,649 / 221,554 (17%) | 869 / 5,242 (17%) | 1,478 / 10,598 (14%) | 1,238 / 8,057 (15%) | 54 / 397 (14%) |
| Low | 75,121 / 220,514 (34%) | 73,140 / 221,554 (33%) | 1,540 / 5,242 (29%) | 1,888 / 10,598 (18%) | 1,688 / 8,057 (21%) | 77 / 397 (19%) |
| Unknown | 3,787 / 220,514 (2%) | 3,876 / 221,554 (2%) | 111 / 5,242 (2%) | 153 / 10,598  (1%) | 152 / 8,057  (2%) | 5 / 397  (1%) |
| Smoking status |  |  |  |  |  |  |
| Current | 27,629 / 220,514 (13%) | 20,133 / 221,554 (9%) | 394 / 5,242 (8%) | 775 / 10,598  (7%) | 557 / 8,057  (7%) | 30 / 397  (8%) |
| Previous | 77,230 / 220,514 (35%) | 75,368 / 221,554 (34%) | 1,719 / 5,242 (33%) | 3,787 / 10,598 (36%) | 2,317 / 8,057 (29%) | 159 / 397 (40%) |
| Never | 114,918 / 220,514 (52%) | 125,281 / 221,554 (57%) | 3,110 / 5,242 (59%) | 6,005 / 10,598 (57%) | 5,147 / 8,057 (64%) | 207 / 397 (52%) |
| Unknown | 737 / 220,514 (0%) | 772 / 221,554 (0%) | 19 / 5,242  (0%) | 31 / 10,598  (0%) | 36 / 8,057  (0%) | 1 / 397  (0%) |
| Physical activity |  |  |  |  |  |  |
| High | 52,700 / 220,514 (24%) | 52,832 / 221,554 (24%) | 1,586 / 5,242 (30%) | 2,958 / 10,598 (28%) | 2,004 / 8,057 (25%) | 120 / 397 (30%) |
| Moderate | 84,985 / 220,514 (39%) | 88,712 / 221,554 (40%) | 1,986 / 5,242 (38%) | 4,606 / 10,598 (43%) | 3,349 / 8,057 (42%) | 177 / 397 (45%) |
| Low | 32,972 / 220,514 (15%) | 30,479 / 221,554 (14%) | 568 / 5,242 (11%) | 1,142 / 10,598 (11%) | 1,087 / 8,057 (13%) | 49 / 397 (12%) |
| Unknown | 49,857 / 220,514 (23%) | 49,531 / 221,554 (22%) | 1,102 / 5,242 (21%) | 1,892 / 10,598 (18%) | 1,617 / 8,057 (20%) | 51 / 397 (13%) |
| Thyroid impairing drugs | 886 / 220,514 (0%) | 711 / 221,554 (0%) | 20 / 5,242  (0%) | 22 / 10,598  (0%) | 27 / 8,057  (0%) | 2 / 397  (1%) |
| *Abbreviation: BMI, body mass index (calculated as weight in kilograms divided by height in meters squared).*  *Categorical variables are expressed as the number of cases divided by the total number of observations (n/N), followed by the percentage in parentheses: n/N (%). Continuous variables are expressed as the arithmetic mean and standard deviation (mean ± (SD)).* | | | | | | |

**Table 3:** Intake of different food types according to 24-hour recall among diet groups within a subsample of 207,011 participants

|  | High meat eaters N = 93,2831 | Low meat eaters N = 100,6991 | Poultry eaters N = 24,131 | Pescatarians N = 59,601 | Vegetarians N = 44,021 | Vegans N = 2,541 |
| --- | --- | --- | --- | --- | --- | --- |
| Dairy | 0.65 (0.88) | 0.62 (0.84) | 0.65 (0.85) | 0.81 (0.88) | 0.94 (0.97) | 0.21 (0.62) |
| Cheese | 0.51 (0.65) | 0.50 (0.64) | 0.53 (0.70) | 0.71 (0.73) | 0.81 (0.80) | 0.17 (0.55) |
| Fish | 0.31 (0.49) | 0.35 (0.49) | 0.47 (0.57) | 0.47 (0.57) | 0.01 (0.11) | 0.01 (0.06) |
| Poultry | 0.34 (0.56) | 0.32 (0.51) | 0.30 (0.49) | 0.01 (0.13) | 0.00 (0.07) | 0.00 (0.06) |
| Red meat | 1.12 (1.03) | 0.72 (0.82) | 0.13 (0.43) | 0.03 (0.24) | 0.01 (0.14) | 0.02 (0.16) |
| Cruciferous vegetables | 0.61 (0.90) | 0.64 (0.92) | 0.76 (1.03) | 0.69 (0.96) | 0.68 (0.99) | 1.28 (1.36) |
| Broccoli | 0.22 (0.42) | 0.24 (0.43) | 0.29 (0.47) | 0.26 (0.46) | 0.25 (0.45) | 0.42 (0.58) |
| Cabbage | 0.14 (0.36) | 0.14 (0.36) | 0.15 (0.37) | 0.15 (0.38) | 0.15 (0.38) | 0.34 (0.61) |
| Turnip | 0.04 (0.19) | 0.04 (0.18) | 0.04 (0.19) | 0.03 (0.16) | 0.04 (0.19) | 0.06 (0.22) |
| Watercress | 0.03 (0.17) | 0.05 (0.20) | 0.09 (0.31) | 0.08 (0.25) | 0.07 (0.25) | 0.12 (0.34) |
| Cauliflower | 0.13 (0.34) | 0.12 (0.34) | 0.13 (0.35) | 0.13 (0.35) | 0.14 (0.37) | 0.23 (0.52) |
| Sprouts | 0.05 (0.24) | 0.05 (0.23) | 0.06 (0.27) | 0.05 (0.25) | 0.05 (0.24) | 0.11 (0.42) |
| *Variables are expressed as the arithmetic mean and standard deviation (mean (±SD))* | | | | | | |

**Table 4**: Average micronutrient intake across dietary groups with data from first FFQ (n = 207,011)

|  | High meat eaters N = 93,283 | Low meat eaters N = 100,699 | Poultry eaters N = 2,413 | Pescatarians N = 5,960 | Vegetarians N = 4,402 | Vegans N = 254 |
| --- | --- | --- | --- | --- | --- | --- |
| Iodine average (in µg) | 217 (105) | 207 (102) | 204 (107) | 208 (107) | 165 (68) | 93 (45) |
| Selenium average (in µg) | 54 (25) | 52 (24) | 55 (28) | 52 (26) | 35 (15) | 35 (14) |
| Iron average (in mg) | 12.7 (4.0) | 12.0 (3.8) | 12.1 (4.3) | 12.9 (4.2) | 13.1 (4.6) | 15.2 (5.9) |
| Haem iron (in mg) | 0.72 (0.53) | 0.57 (0.46) | 0.32 (0.36) | 0.17 (0.22) | 0.02 (0.09) | 0.02 (0.08) |
| Vit B12 average (in µg) | 6.47 (3.46) | 6.05 (3.25) | 5.75 (3.75) | 5.63 (3.12) | 3.77 (1.98) | 2.13 (1.55) |
| Copper average (in mg) | 1.40 (0.54) | 1.36 (0.52) | 1.44 (0.62) | 1.51 (0.51) | 1.55 (0.57) | 1.91 (0.75) |
| Zinc average (in mg) | 10.3 (3.5) | 9.3 (3.2) | 8.4 (3.0) | 8.6 (2.8) | 9.0 (3.3) | 8.2 (3.1) |
| *Variables are expressed as the arithmetic mean and standard deviation (mean ± (SD))*  *Abbreviation: Vit B12, Vitamin B12.* | | | | | | |

**Table 5**: Iodine intake on whether individuals met or did not meet the recommended intake threshold of over 150 μg/day among a subsample of 207,011

|  | Iodine below 150 μg/day | Iodine above 150 μg/day |
| --- | --- | --- |
| High meat eaters | 22,803 (24.2 %) | 71,519 (75.8 %) |
| Low meat eaters | 29,175 (28.6 %) | 72,900 (71.4 %) |
| Poultry eaters | 806 (32.9 %) | 1,643 (67.1 %) |
| Pescatarians | 1,855 (30.7 %) | 4,184 (69.3 %) |
| Vegetarians | 1,985 (44.4 %) | 2,489 (55.6 %) |
| Vegans | 236 (92.2 %) | 20 (7.81 %) |
| *Variables are expressed as frequencies and percentages (n (%))* | | |

**Table 6**: Demographic characteristics of prevalent hypothyroid cases

|  | **Characteristics** | **Non-cases (N = 466,362)** | **Cases (N =** **27,782)** |
| --- | --- | --- | --- |
| Diet | High meat eaters | 220,514 / 466,362 (47.3%) | 11,263 / 27,782 (40.5%) |
|  | Low meat eaters | 221,554 / 466,362 (47.5%) | 14,811 / 27,782 (53.3%) |
|  | Poultry eaters | 5,242 / 466,362 (1.1%) | 415 / 27,782 (1.5%) |
|  | Pescatarians | 10,598 / 466,362 (2.3%) | 682 / 27,782 (2.5%) |
|  | Vegetarians | 8,057 / 466,362 (1.7%) | 589 / 27,782 (2.1%) |
|  | Vegans | 397 / 466,362 (0.1%) | 22 / 27,782 (0.1%) |
| Sex | Female | 245,810 / 466,362 (52.7%) | 23,338 / 27,782 (84.0%) |
|  | Male | 220,552 / 466,362 (47.3%) | 4,444 / 27,782 (16.0%) |
| Age | | 56.49 (8.09) | 56.41 (8.11) |
| Ethnicity | White | 440,650 / 466,361 (94.5%) | 26,465 / 27,782 (95.3%) |
|  | Mixed | 2,761 / 466,361 (0.6%) | 132 / 27,782 (0.5%) |
|  | Asian | 10,054 / 466,361 (2.2%) | 686 / 27,782 (2.5%) |
|  | Black | 7,268 / 466,361 (1.6%) | 209 / 27,782 (0.8%) |
|  | Unknown | 5,629 / 466,361 (1.2%) | 290 / 27,782 (1.0%) |
| BMI (kg/m^2^) |  | 27.35 (4.74) | 28.52 (5.49) |
|  | Unknown | 2,440 | 144 |
| Average Income | Greater than 100,000 | 22,085 / 466,362 (4.7%) | 801 / 27,782 (2.9%) |
|  | 52,000 to 100,000 | 82,604 / 466,362 (17.7%) | 3,334 / 27,782 (12.0%) |
|  | 31,000 to 51,999 | 104,910 / 466,362 (22.5%) | 5,125 / 27,782 (18.4%) |
|  | 18,000 to 30,999 | 100,651 / 466,362 (21.6%) | 6,310 / 27,782 (22.7%) |
|  | Less than 18,000 | 88,193 / 466,362 (18.9%) | 6,938 / 27,782 (25.0%) |
|  | Unknown | 67,919 / 466,362 (14.6%) | 5,274 / 27,782 (19.0%) |
| Education | High | 220,046 / 466,362 (47.2%) | 11,707 / 27,782 (42.1%) |
|  | Medium | 84,778 / 466,362 (18.2%) | 4,390 / 27,782 (15.8%) |
|  | Low | 153,454 / 466,362 (32.9%) | 11,192 / 27,782 (40.3%) |
|  | Unknown | 8,084 / 466,362 (1.7%) | 493 / 27,782 (1.8%) |
| Smoking status | Current | 49,518 / 466,362 (10.6%) | 2,297 / 27,782 (8.3%) |
|  | Previous | 160,580 / 466,362 (34.4%) | 10,412 / 27,782 (37.5%) |
|  | Never | 254,668 / 466,362 (54.6%) | 14,949 / 27,782 (53.8%) |
|  | Unknown | 1,596 / 466,362 (0.3%) | 124 / 27,782 (0.4%) |
| Physical activity | High | 112,200 / 466,362 (24.1%) | 5,963 / 27,782 (21.5%) |
|  | Moderate | 183,815 / 466,362 (39.4%) | 10,070 / 27,782 (36.2%) |
|  | Low | 66,297 / 466,362 (14.2%) | 4,037 / 27,782 (14.5%) |
|  | Unknown | 104,050 / 466,362 (22.3%) | 7,712 / 27,782 (27.8%) |
| Thyroid impairing drugs |  | 1,668 / 466,362 (0.4%) | 293 / 27,782 (1.1%) |
| *Abbreviation: BMI, body mass index (calculated as weight in kilograms divided by height in meters squared).*  *Categorical variables are expressed as the number of cases divided by the total number of observations (n/N), followed by the percentage in parentheses: n/N (%). Continuous variables are expressed as the arithmetic mean and standard deviation (mean ± (SD)).* | | | |

**Figure 4:** Hazard ratios (HR) and 95% Confidence intervals (95% CI) between diet groups and the risk of hypothyroidism with all hypothyroidism cases and cases only due to potential low iodine intakes.


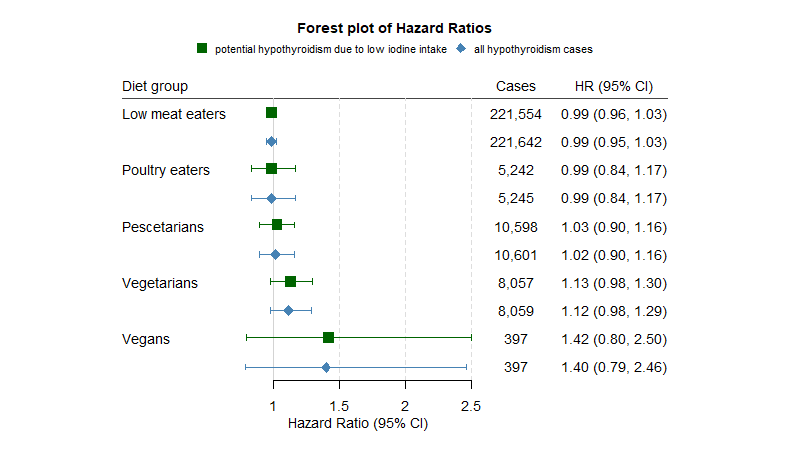


*All models adjusted for sex, age, income, education, ethnicity, thyroid impairing medication and smoking status.*
